# Synthetic bacterial consortium for degradation of plastic pyrolysis oil waste: experimental optimization and neural network modeling

**DOI:** 10.3389/fmicb.2026.1817874

**Published:** 2026-07-03

**Authors:** Yunpu Jia, Jingxi Dou, Hendrik Ballerstedt, Jianmin Xing, Lars M. Blank

**Affiliations:** 1 State Key Laboratory of Petroleum Molecular and Process Engineering, Institute of Process Engineering, Chinese Academy of Sciences, Beijing, China; 2 College of Chemical Engineering, University of Chinese Academy of Sciences, Beijing, China; 3 State Key Laboratory of Multiphase Complex Systems, Institute of Process Engineering, Chinese Academy of Sciences, Beijing, China; 4 WSS Center Catalaix, Institute of Applied Microbiology (iAMB), Aachen Biology and Biotechnology (ABBt), RWTH Aachen University, Aachen, Germany

**Keywords:** bacterial consortium, bioremediation, dearomatization, neural network model, plastic degradation, strain compounding

## Abstract

**Introduction:**

The plastic crisis is omnipresent, ranging from plastic littering to microplastics found in every niche of the planet, including the human body. To achieve higher recycling quotas, especially for mixed plastic waste, pyrolysis is a viable option. However, plastic pyrolysis oil waste (PPOW) poses significant challenges for valorization due to its extreme molecular heterogeneity.

**Methods:**

To reduce this molecular heterogeneity, we artificially compounded, monitored, and optimized a bacterial consortium capable of tolerating organic pollutants and utilizing them as carbon and energy sources. Additionally, a back-propagation (BP) neural network was applied to evaluate O_2_ consumption as an indicator of microbial activity and to build a predictive model for process optimization.

**Results:**

The primary constituents of the PPOW were alkanes and ε-caprolactam. Within 7 days, the bacterial community demonstrated remarkable efficacy, degrading alkanes (C10–C28) at rates of 71–100% and achieving complete removal of ε-caprolactam (8,032 mg/mL) and naphthalene. The AI model predicted O_2_ consumption with high accuracy (R^2^ > 0.99).

**Discussion:**

This AI-driven approach significantly reduces experimental iterations and operational costs for future process optimization. These results are discussed in the context of a developing open circular plastic economy.

## Graphical Abstract

**Figure:**
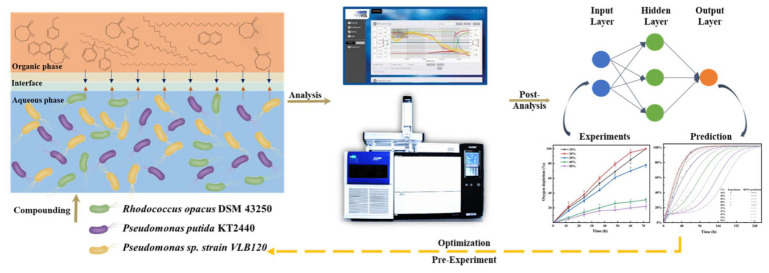

## Highlights

- Synthetic bacterial consortium are used to remove plastic pyrolysis oil waste
- Majority of pollutants can be cleared in optimized biphasic reaction system
- The biodegradation process can be monitored in a real-time bioprocess software
- Neural network techniques are used to model and predict the removal process

## 1 Introduction

Plastics have become the most relevant materials in modern societies. Worldwide plastic production is anticipated to double within the next two decades and is projected to nearly quadruple by 2050 (Lebreton and Andrady, 2019). Given the limitations of mechanical recycling, non-recovered plastics cause severe pollution and threaten life of animals and humans. The necessity for a “post-consumer plastics economy” has become evident and is influencing political and business choices (He et al., 2026). The key to achieving a circular economy is to identify appropriate technologies. Reprocessing plastics without compromising function and value necessitates a paradigm shift in our understanding of plastic recycling. The hydrocarbon resource targeted for recovery has to demonstrate potential for diverse and high-value applications (Wang et al., 2022), a concept also known as loop-recycling. This ranges from the conversion of plastics into highly efficient fuels and oil products for the polymer industry.

CARBOLIQ GmbH (Remscheid, Germany) has demonstrated the ability to transform mixed plastic waste into a liquid resource by applying a one-stage conversion technology, catalytic tribochemical conversion (CTC). This technology integrates thermal, catalytic, and mechanochemical (tribochemical) mechanisms in a direct liquefaction process. CTC operates under mild conditions at atmospheric pressure and process temperatures of around 400°C. The solid input material is continuously fed to the hot liquid system enriched with a specialized catalyst capable of cleaving polymer chains. The oiled plastic is separated by distillation with obtainment of a plastic pyrolysis oil waste (PPOW) fraction. This fraction can be difficult to valorize, as it constitutes a mixture of molecules with varying chemical and physical properties.

Considering the complexity of the substrate, we propose a strategy to treat this complex fraction using artificially compounded strains in a synthetic microbial consortium (Deng et al., 2025; Zhou and Zeeshan Ul Haq, 2025). *Pseudomonads* play a crucial role in element cycling (Palleroni, 1984) and exhibit significant potential for bioremediation. They boast a diverse repertoire of pathways for degrading various non-natural and non-inherent pollutants, particularly aromatic organics (Esikova et al., 2023; He et al., 2026; Vetal et al., 2023; Zhou and Zeeshan Ul Haq, 2025). Among these, *Pseudomonas putida* KT2440 (Bagdasarian et al., 1981; Bleem et al., 2024) stands out as the most extensively characterized saprophytic *Pseudomonas*, maintaining its ability to thrive and perform essential functions in the environment. It exhibits catabolic potential toward a wide range of natural aromatic compounds. *Pseudomonas* sp. VLB120 (Guzik et al., 2021; Zhou and Zeeshan Ul Haq, 2025) underwent examination for its inherent tolerance to toxic compounds like toluene and styrene, with genomic analyses identifying homologs of known solvent-tolerance genes. *Rhodococcus* bacteria are widely acknowledged for their significant potential in bioremediation and have been successfully employed for the removal of contaminants from soil, water, and air (Deng et al., 2025). *Rhodococcus opacus* DSM 43250 is a Gram-positive bacterium that was first isolated from soil. Its prominent metabolic characteristics encompass limited catabolic repression by readily available carbon sources (Anokhina et al., 2025) and adaptable biodegradation pathways for both sugars and aromatics (Henson et al., 2018; Liu et al., 2018).

In this study, the microbial consortium consisted of three bacterial strains, *Rhodococcus opacus* DSM 43250, *Pseudomonas putida* KT2440, and *Pseudomonas* sp. VLB120. We hypothesized that this consortium could synergistically degrade PPOW in a biphasic system. The PPOW was characterized and used as a carbon and energy source for the synthetic microbial consortium within a 2-nonanone-based biphasic system to enhance substrate bioavailability. The degradation of PPOW by bacterial consortium was evaluated by analyzing degradation ratios of each of its major components following process enhancement. To characterize the growth dynamics of the consortium, a three-layer back-propagation (BP) neural network was employed to model the experimental data and predict oxygen consumption in subsequent experiments. The novelty of this work lies in the utilization of CARBOLIQ derived PPOW, the use of 2-nonanone as the organic phase, the AI driven O_2_ prediction model, and the complete removal of ε-caprolactam. This integrated approach achieves significant PPOW degradation efficiency while remaining cost-effective. Furthermore, this study addresses multiple SDGs by converting plastic waste into valuable biomass, providing an alternative to CO_2_ emitting incineration, preventing marine microplastic pollution, and integrating AI driven process optimization (The Global Goals, 2015a; The Global Goals, 2015b; The Global Goals, 2015c; The Global Goals, 2015d). Thus, this bioprocess thus represents a practical step toward the UN Sustainable Development Goals.

### 2 Materials and methods

#### 2.1 Chemicals

PPOW was supplied by CARBOLIQ GmbH (Remscheid, Germany). Alkane standard solution was purchased from Merck (Darmstadt, Germany). Water was purified using a Milli-Q Academic System (18.2 MΩ cm; 0.22-μm filter) (Millipore, Molsheim, France). Other chemicals used in this work were obtained from Carl Roth (Karlsruhe, Germany) and Sigma-Aldrich (St. Louis, MO, United States), unless stated otherwise.

### 2.2 Components of culture medium

The precultures were made in the Delft mineral salt medium (MSM). The final medium composition per liter was 3.88 g of K_2_HPO_4_, 1.63 g of NaH_2_PO_4_, 2.00 g of (NH_4_)_2_SO_4_, 0.1 g of MgCl_2_ ⋅ 6 H_2_O, 10 mg of EDTA, 2 mg of ZnSO_4_ ⋅ 7 H_2_O, 1 mg of CaCl_2_ ⋅ 2 H_2_O, 5 mg of FeSO_4_ ⋅ 7 H_2_O, 0.2 mg of Na_2_MoO_4_ ⋅ 2 H_2_O, 0.2 mg of CuSO_4_ ⋅ 5 H_2_O, 0.4 mg of CoCl_2_ ⋅ 6H_2_O, and 1 mg of MnCl_2_ ⋅ 2 H_2_O, supplemented with 20 mM sodium benzoate as carbon source. Precultures of single strains consisted of 10 mL medium in 50 mL Erlenmeyer flasks. Prior to inoculation, all strains were pre-cultured for 24 h at 30°C and 200 rpm. Cells were harvested in the late exponential growth phase (OD_600_ = 1.0).

### 2.3 Bacteria

*Rhodococcus opacus* DSM 43250, *Pseudomonas putida* KT2440, and *Pseudomonas* sp. VLB120 were stored in the strain collection of the Institute of Applied Microbiology (RWTH Aachen University, Aachen, Germany).

### 2.4 Biodegradation reaction

For the two-phase reaction, cells were cultured at the specified temperature in a modified MSM (Demling et al., 2020). The standard phosphate buffer capacity of the medium was increased three-fold to counteract acidification during *Pseudomonas* cultivation. The final aqueous culture medium composition per liter was: 11.64 g of K_2_HPO_4_, 4.89 g of NaH_2_PO_4_, 2.00 g of (NH_4_)_2_SO_4_, 0.1 g of MgCl_2_ ⋅ 6H_2_O, 10 mg of EDTA, 2 mg of ZnSO_4_ ⋅ 7H_2_O, 1 mg of CaCl_2_ ⋅ 2H_2_O, 5 mg of FeSO_4_ ⋅ 7H_2_O, 0.2 mg of Na_2_MoO_4_ ⋅ 2H_2_O, 0.2 mg of CuSO_4_ ⋅ 5H_2_O, 0.4 mg of CoCl_2_ ⋅ 6H_2_O, and 1 mg of MnCl_2_ ⋅ 2H_2_O. Ethyl decanoate or 2-nonanone was selected as the organic phase in the reaction system, which contains PPOW as the main carbon and energy source, with 0.05% (w/v) yeast extract added to promote cell growth.

To investigate the O_2_ consumption rate under various cultivation conditions, 200 mL anaerobic serum bottles with aluminum caps were used. The total volume of the biphasic medium was 20 mL and aeration was provided solely via the headspace. The cell densities of the individual strains were quantified by measuring the optical density at 600 nm (OD_600_). These experimental conditions were also applied to CO_2_ production measurements, except that the reaction volume consisted of 50 mL medium in a 1 L Schott-Duran bottle. The inoculum was prepared by mixing the three strains at a specified ratio and centrifuging the suspension at 5,000 rpm for 10 min. The resulting pellet was washed twice with sterile MSM to remove residual benzoate. The final cell density was adjusted to an OD600 of 1.0 using sterile medium and a 10% (v/v) inoculum was transferred into the biphasic reaction system. To distinguish between biotic degradation and abiotic loss (e.g., volatilization), heat-killed controls were prepared by autoclaving the inoculum (121°C, 20 min) and incubating under identical conditions. Net biodegradation efficiency was calculated by subtracting the removal percentage observed in abiotic controls from that in biotic samples to account for losses due to volatilization and adsorption.

### 2.5 Extraction of residual PPOW

Residual PPOW was sampled by liquid-liquid extraction. Briefly, broth culture (50 mL) was extracted twice with a five-volumes of n-hexane. After removing the aqueous phase using a separating funnel, the residual concentration of PPOW was determined by gas chromatography equipped with a flame ionization detector (GC-FID). To validate the liquid-liquid extraction procedure, recovery experiments were conducted by spiking sterile media with known concentrations of target compounds (ε-caprolactam and alkanes). The samples were processed using the standard extraction protocol, and recovery rates were calculated as the ratio of measured to spiked concentrations. Average recoveries ranged from 95 to 105%, with relative standard deviations (RSD) below 5%, confirming the reliability of the method. Data were analyzed using OriginPro. Results are expressed as mean ± standard deviation (SD) from three independent replicates. One-way ANOVA followed by Tukey’s multiple comparison test was employed to evaluate differences between compounds. Statistical significance was set at *p* < 0.05.

### 2.6 Analytical methods

#### 2.6.1 CO_2_ measurement

A BCP-CO_2_ gas analyzer coupled with a bioprocess software (BlueSens Gas Sensor GmbH, Herten, Germany) was used for continuous, real-time *in-situ* CO_2_ analysis. The CO_2_ concentration was monitored via infrared light, which is attenuated by the analyte gas and reflected onto the sensor’s detector unit. The sensor was airtightly affixed to the opening of a 1 L static Schott-Duran bottle used as the culture vessel. Measurements were conducted under static conditions for up to 1 week, with the maximum duration constrained by oxygen availability for microbial metabolism.

#### 2.6.2 Gas-chromatography to assess O_2_ depletion

The O_2_ was measured by an SRI 8610C Multi-detector Gas Chromatograph (GC) coupled with a helium ionization detector (HID). A HayeSepD Pre-column (2 mm × 2 m) and a main column (Molsieve 13x) were used to separate O_2_ from the gas mixture. O_2_ content was quantified using a standard curve. The column was connected to a thermal conductivity detector (TCD, 157°C) at higher concentrations and a HID (100 V, 204°C) at lower concentrations. Helium carrier gas was supplied at 48 mL/min, and the column was operated isothermally at 60°C.

#### 2.6.3 Gas chromatography-mass spectrometry (GC-MS)

The original sample and alkane standard solution were analyzed using GC-MS after filtration and a 10-fold dilution with n-hexane. The GC-MS analysis was performed using a Shimadzu GC-MS QP2010 Ultra system equipped with a 30 m Rtx-5MS column (internal diameter, 0.25 mm, film thickness, 0.25 μm). Helium served as the carrier gas at a flow rate of 3 mL/min, and 1 μL of the sample was injected in splitless mode. The GC oven temperature was programmed as follows: initial temperature of 50°C, ramped at 6°C/min to 280°C, and held for 5 min.

#### 2.6.4 GC-FID

Hexane extracts (1.0 μL) of PPOW were analyzed using a Hewlett Packard 5890 Series II GC equipped with a flame ionization detector (FID) and 30 m long ZB-WAX column (internal diameter, 0.25 mm, film thickness, 0.25 μm). Nitrogen was used as the carrier gas. The injector and detector temperatures were maintained at 200 and 290°C, respectively. The GC oven temperature was programmed as follows: an initial temperature of 40°C held for 1 min, ramped at 1°C/min to 50°C, followed by a rapid increase at 40°C/min to 200°C, and held for a total run time of 35 min. Calibration curves were plotted using standard solutions of alkanes (C10-C28), ε-caprolactam, ethylbenzol, cumene, naphthalin and toluene. The method exhibited excellent linearity over the concentration range of 10–1,800 μg/mL with correlation coefficients (*R*^2^) > 0.99. The limits of detection (LOD) and quantification (LOQ) were determined based on signal-to-noise ratios of 3:1 and 10:1, respectively.

#### 2.6.5 Neural network model

A three-layer back-propagation neural network with an input-hidden-output architecture of 2-5-1 nodes was constructed. The input variables were time (hours) and one of the three operational parameters (temperature, organic-to-aqueous phase ratio, or PPOW content in the second phase). The output variable was O_2_ depletion (dimensionless, normalized to initial concentration). The hidden layer used a sigmoid activation function *f(x)* = 1/(1 + *e^–*x*^*), while the output layer used a linear activation function. All input and output data were normalized to the range [0, 1] using min-max scaling: *x*_*norm*_ = (*x* − *x*_*min*_)/(*x*_*max*_–*x*_*min*_). The dataset comprised 120 experimental samples and was randomly split into training (70%) and testing (30%) sets. To ensure model robustness and address the need for rigorous validation beyond a simple split, a five-fold cross-validation was additionally performed on the training dataset. Hyperparameter tuning was conducted via grid search, optimizing the number of hidden nodes (tested range: 3–7), learning rate, and L2 regularization weight. Overfitting was mitigated using early stopping with a patience of 500 epochs and L2 regularization with λ = 0.01. The model performance was evaluated using the coefficient of determination (*R*^2^) and mean squared error (MSE).

#### 2.6.6 Statistical analysis

All experiments were performed in triplicate (*n* = 3), and data are presented as mean ± standard deviation (SD). Statistical analyses were conducted using *Python*3.12. One-way analysis of variance (ANOVA) was employed to determine the statistical significance of the differences in O_2_ depletion rates across the various optimization conditions (temperature, organic-to-aqueous phase ratio, and PPOW content). For factors yielding a significant overall effect (*p* < 0.05), Tukey’s *post-hoc* test was performed for multiple pairwise comparisons to identify specific differences between the optimal condition and other tested parameters. A *p*-value of < 0.05 was considered statistically significant.

## 3 Results and discussion

### 3.1 Substrate characterization

**FIGURE 1:**
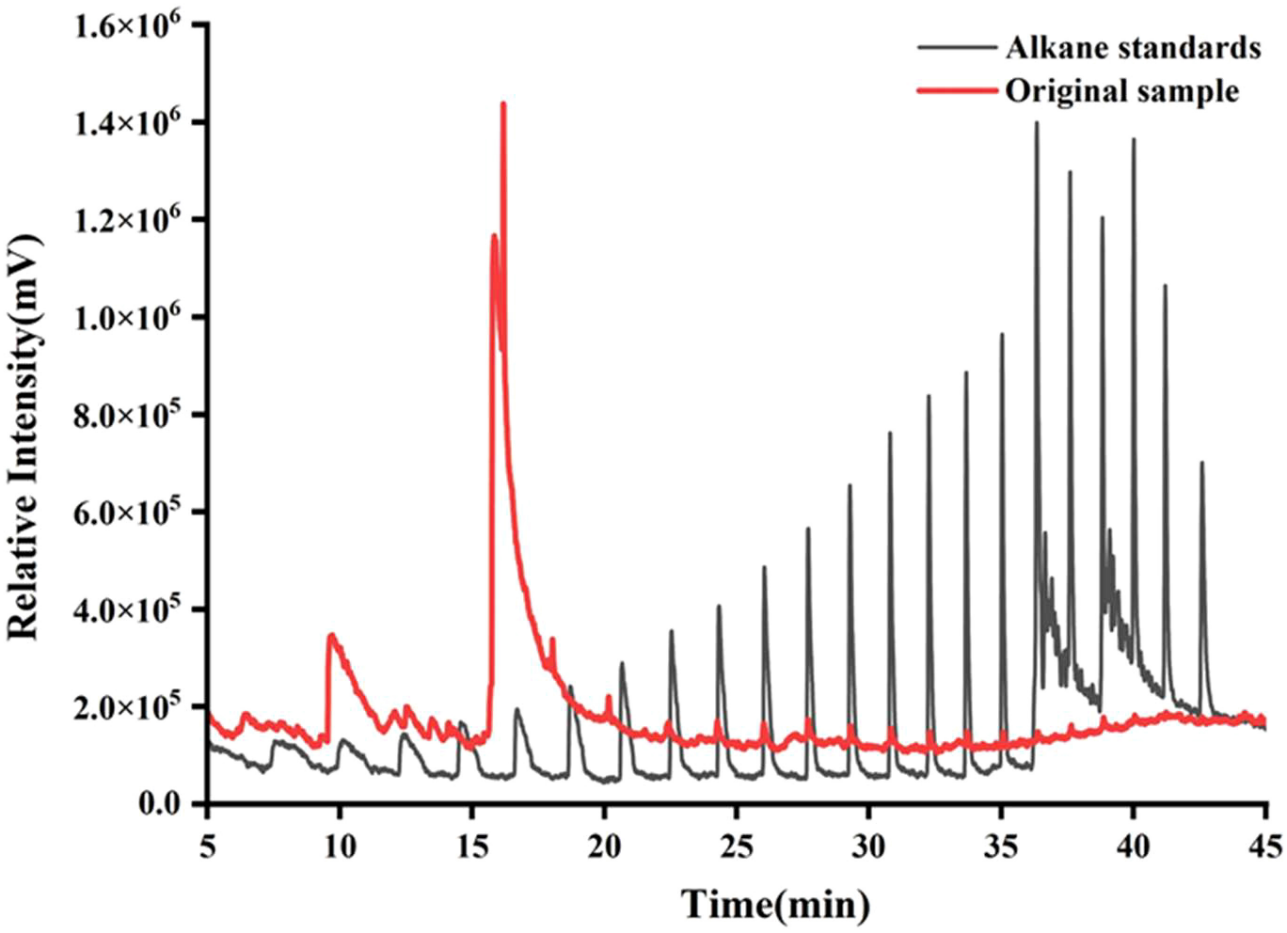
Substrate characterization by GC-MS. The plastic pyrolysis oil waste (PPOW, red) was filtered and diluted 5 times by n-hexane and was compared against an alkane standard.

GC-MS and GC-FID were used to determine the composition of the PPOW. Given that the end product of the plastic pyrolysis process consists of alkanes of various chain lengths, it was hypothesized that the sample would contain a substantial quantity of alkanes. Based on the raw material used, the sample was also expected to contain various aromatic compounds. From the result of GC-MS in Figure 1, the predominant peaks in the sample were identified as alkanes, and the mass spectrometry results revealed that the non-alkane substances with elevated contents included cumene, toluol, ethylbenzol, naphthalin, and ε-caprolactam. Notably, ε-caprolactam exhibited the highest content (8,032 μg/mL), far exceeding that of alkanes. We verified this result using GC-FID and quantified the content of these five substances (Supplementary Figures 1, 2 and Supplementary Table 1).

### 3.2 Optimization of biotic conditions and consortium synergy

**FIGURE 2:**
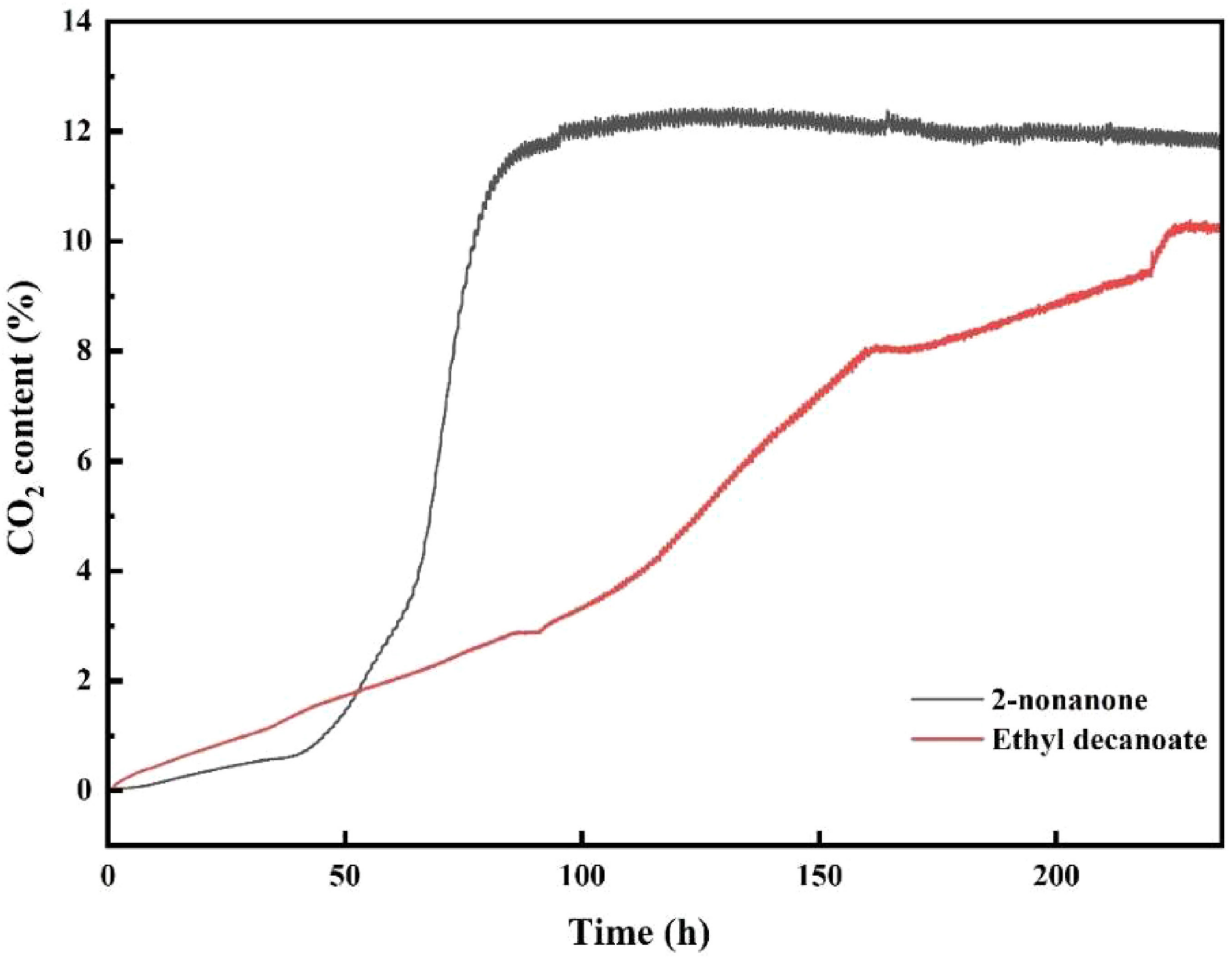
Comparison of 2-nonanone and ethyl decanoate as the second organic phase. CO_2_ release was measured online during PPOW biodegradation in the two phase system.

**FIGURE 3:**
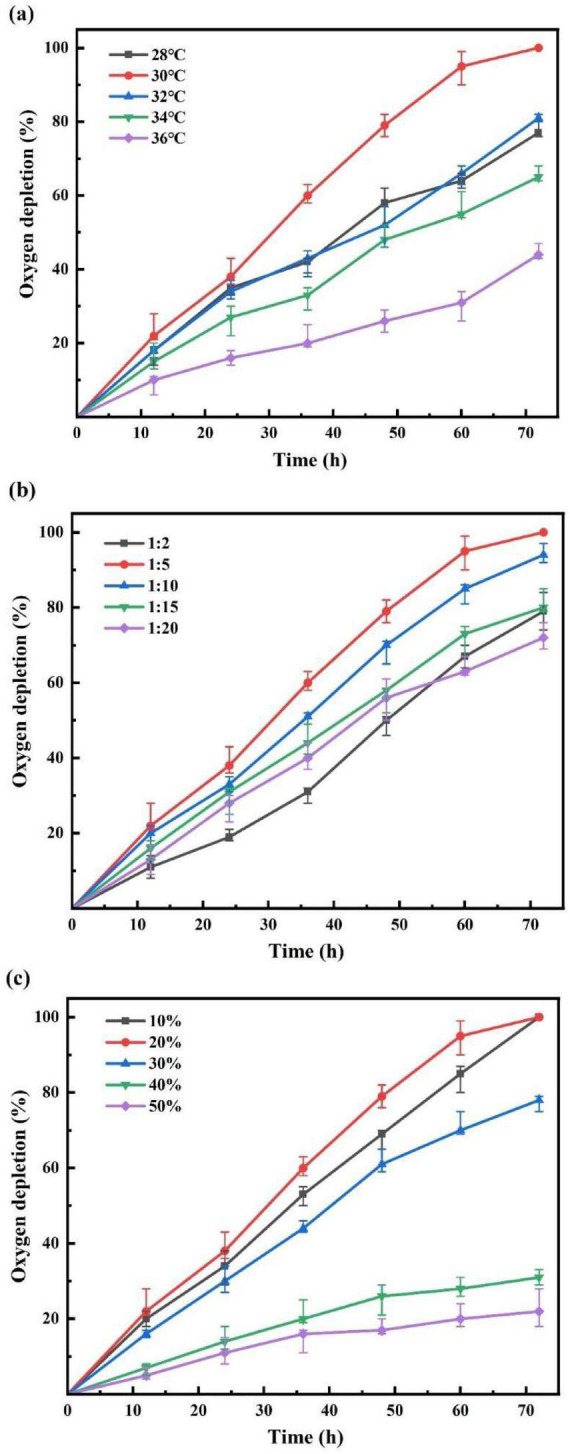
The rate of O_2_ depletion under different growth conditions of the synthetic consortium. **(a)** Temperature, **(b)** organic:aqueous phase ratio, and **(c)** PPOW content (%V) in the second phase.

Given the heterogeneous nature of PPOW, which contains both aliphatic hydrocarbons and toxic aromatic compounds, a single microbial strain is unlikely to possess the complete metabolic repertoire required for its complete degradation. Therefore, we rationally designed a synthetic consortium based on the principle of metabolic division of labor. Biodegradation of PPOW by a synthetic bacterial community composed of *Rhodococcus opacus* DSM 43250, *Pseudomonas putida* KT2440 and *Pseudomonas* sp. VLB120 was investigated in an organic-aqueous two-phase system. To mitigate the toxic effects of PPOW on strains, previouly used second phase compounds, namely 2-nonanone (Grütering et al., 2024) and ethyl decanoate (Demling et al., 2020), served as a second organic phase each. Their role was to reduce the concentration of the organic components in the aqueous phase while serving as a substrate reservoir. A BCP-CO_2_ gas analyzers were used for online real-time CO_2_ analysis (Surger and Blank, 2022). The optimal second phase was determined by comparing the release of CO_2_ from the two-phase reaction system. The experiments comparing the two organic phases (Figure 2) were conducted at 30°C with an organic phase to aqueous phase ratio of 1:5, a PPOW content of 20% (v/v) in the organic phase, and an equal strain ratio of 1:1:1 for *Rhodococcus opacus* DSM 43250, *Pseudomonas putida* KT2440, and *Pseudomonas* sp. VLB120. For the optimization of temperature, phase ratio, and PPOW content (Figure 3), the same equal strain ratio was used unless otherwise specified. When using 2-nonanone as the second phase, CO_2_ exhibited a significantly higher release in a shorter period compared to that observed with ethyl decanoate. This suggests that when 2-nonanone was used as the second phase, the bacterial community exhibits a higher metabolic activity, and was chosen here for subsequent experiments. 2-Nonanone was chosen over ethyl decanoate due to its higher log *P*-value (3.14 vs. 2.85), indicating better retention of toxic compounds in the organic phase (Grütering et al., 2024), and lower microbial inhibition (IC50 > 20% v/v vs. 15% for ethyl decanoate).

Afterward, we investigated other factors influencing the metabolic activity of the bacterial consortium and determined the optimal culture conditions based on these findings (Figure 3). The optimal growth temperature for the three strains in the bacterial consortium was found to be 30°C. However, it is crucial to note that the optimum temperature for the growth and metabolism of strains is typically not uniform (Bagdasarian et al., 1981; Gurudiwan et al., 2025). Some microorganisms may display a faster growth rate at lower temperatures, yet their metabolic activity could be more pronounced at higher temperatures. In addition to monitoring the CO_2_ release rate, assessing O_2_ consumption is an equally vital as a key indicator of microbial metabolic activity (Casida, 1977; Gurudiwan et al., 2025; Zeuthen, 1953; Zhou and Zeeshan Ul Haq, 2025). Therefore, to simplify the experiments, all optimization trials measured the O_2_ consumption at different time points under dynamic shaking flask cultivation conditions. The results in Figure 3a indicate that the optimal temperature for the bacterial consortium’s utilization of organic substances in PPOW aligns with the growth temperature. Extreme temperatures, whether high or low, proved unfavorable for the absorption and utilization of carbon sources in PPOW. This could be attributed to the impact of temperature on the metabolic activity of enzymes within the bacterial consortium. Additionally, temperature can influence the solubility of O_2_ in the biphasic reaction solution, thereby directly affecting the microbial utilization of O_2_. In a biphasic reaction system, the ratio of the organic phase to the aqueous phase is undoubtedly a crucial factor influencing the reaction rate by affecting the concentration of organic matter exposed to the bacterial consortium. Maximum degradation was achieved at an organic-to-aqueous ratio of 1:5. Further increasing the ratio did not lead to a higher degradation rate. Therefore, a 1:5 ratio was selected for subsequent studies. Excessive concentrations of PPOW can exert a toxic effect on the microbial community, while low concentrations may not fully exploit the degradative capabilities of the bacterial consortium. The findings suggest that when PPOW constitutes 20% (v/v) of the second phase, O_2_ depletion in the closed system occurs most rapidly, implying optimal removal efficiency (Figure 3c). Statistical significance of the differences between these optimization conditions (temperature, phase ratio, and PPOW content) was evaluated using one-way ANOVA followed by Tukey’s *post-hoc* test (*p* < 0.05), confirming that the selected optimal conditions were significantly superior to the alternative tested parameters (see Supplementary Table 3).

**FIGURE 4:**
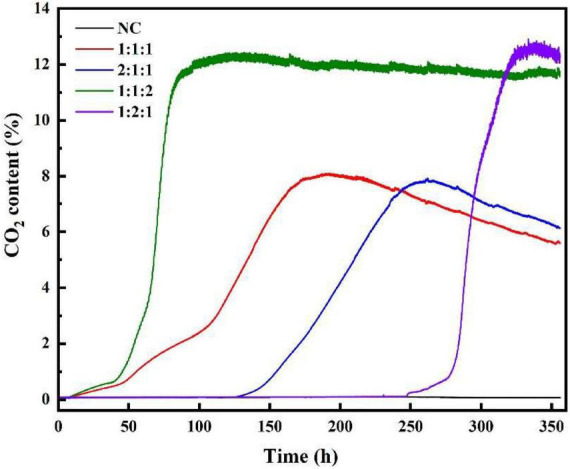
Evaluating bacterial consortium composition on CO_2_ release. NC, negative control groups without adding any bacteria.

In addition, we explored the impact of varying strain compositions within the bacterial consortium on the removal of PPOW (Figure 4). Different from the previous experimental conditions, the microbial community displayed maximum metabolic activity when the ratio of *R. opacus* DSM 43250, *P. putida* KT2440, *Pseudomonas* sp. VLB120 was 1:1:2. This specific composition of the bacterial consortium exhibited a distinct kinetic advantage in the reaction system. Although the specific mechanism remains unclear, we infer that the high degradation efficiency achieved in this study stems from rational functional complementarity among the consortium members. *Rhodococcus opacus* DSM 43250 serves as the principal driver for long-chain alkane (C10-C28) degradation, utilizing its potent alkane hydroxylase machinery. In contrast, the Pseudomonas strains are dedicated to aromatic catabolism and stress resistance. Notably, *P. putida* KT2440 targets aromatic fractions, while *Pseudomonas* sp. VLB120 confers significant solvent tolerance, alleviating the inhibitory effects of toxic intermediates. This division of labor, which integrates aliphatic oxidation with aromatic detoxification, accounts for the consortium’s superior efficacy compared to that of its individual constituents (Schwanemann et al., 2025).

### 3.3 Degradation performance of key pollutants

**FIGURE 5:**
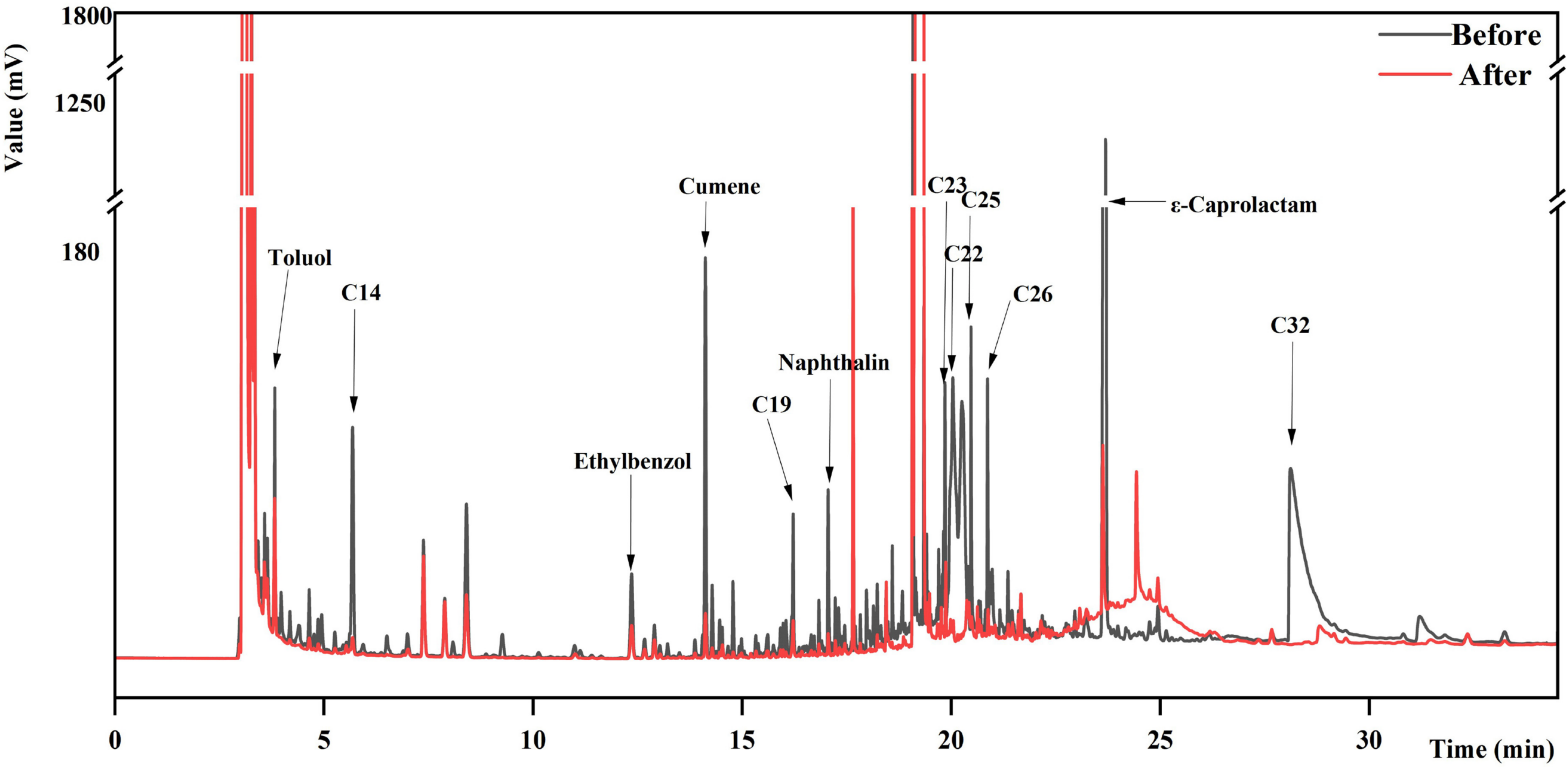
Substrate uptake analysis. GC-FID chromatograms of original PPOW (black) and PPOW used as carbon and energy source by the synthetic bacterial consortium under optimal conditions (red) after 7 days of incubation. Peaks for key compounds are labeled.

**FIGURE 6:**
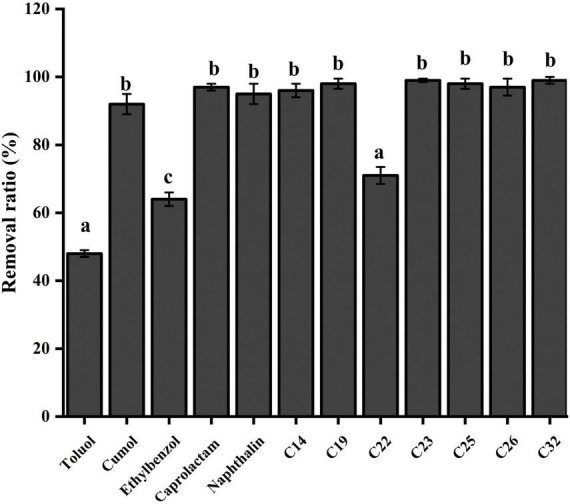
Removal ratios of major substances from PPOW after 7 days of treatment under optimal conditions. Each error bar represents the standard deviation of three replicate experiments (*n* = 3). Different letters above the bars indicate significant differences (*p* < 0.05).

Based on the above discussion, the subsequent long-term studies were carried out to investigate the degradation of aromatic and aliphatic compounds present in PPOW under the following conditions: 30°C, an organic**-**to-aqueous phase ratio of 1:5, a ratio of *R. opacus* DSM 43250: *P. putida* KT2440: *Pseudomonas* sp. VLB120 of 1:1:2, and 20% (v/v) PPOW content in the organic phase. GC-FID was employed to detect changes in PPOW before and after degradation, and to calculate the removal rate (Figure 5). The percentage degradation of several key components present in the PPOW after 7 days of treatment by the bacterial consortium in the biphasic reaction system is shown in Figure 6. With the exception of toluol, the removal rates for more than a dozen substances with relatively high concentrations in the waste all exceeded 50%, with some achieving complete removal after 7 days of biotreatment by the synthetic bacterial community. The low toluene degradation rate (48 ± 1%, *p* < 0.001) may be attributed to competitive inhibition exerted by the high concentration of ε-caprolactam (8,032 μg/mL) present in the PPOW, which may repress the metabolic pathways responsible for toluene catabolism in *P. putida* KT2440. Additionally, the solvent stress response triggered by the biphasic system might prioritize survival over the degradation of less preferred substrates like toluene (Molina-Santiago et al., 2017). To overcome this limitation, future strategies such as fed-batch cultivation or adaptive laboratory evolution could be considered. A fed-batch approach would maintain sub-inhibitory toluene levels and reduce competitive pressure from ε-caprolactam, potentially shifting the metabolic response of *P. putida* KT2440 from stress survival toward active degradation. Alternatively, ALE could be employed to select for *P. putida* KT2440 variants with enhanced toluene tolerance or catabolic capacity under gradually increasing toluene concentrations, either in monoculture or within the consortium context. These strategies, while beyond the scope of the present study, offer promising routes to further improve the overall degradation efficiency of the synthetic consortium. Supplementary Figure 6 shows a distinct alteration in the color of organic phase in the two-phase system after microbial treatment compared to its pre-treatment state. This color transformation from dark brown to light yellow correlated with 65–100% pollutant removal, providing a rapid visual indicator for the removal of organic substances in the treated PPOW.

### 3.4 Neural network model for O_2_ depletion in two-phase system

As oxygen availability is key for PPOW activation and degradation, we investigated the data using an AI model. A three-layer Back-propagation (BP) neural network, with an input-hidden-output architecture of 2-5-1 nodes was constructed, written as


$$y_{0}=x$$

$$y_{n}=w\cdot f_{n}\,\left(y_{n-1}\right)+b$$

where *x* is the input variable, defined as time and influence factors (temperatures, organic: aqueous phase ratio and PPOW content (%V) in the second phase), and *y_n_* is our excepted prediction (O_2_ depletion). *f_n_* is a sigmoid activation function in *n* layer. *w* and *b* are the parameters of the neural network, namely weight and bias, which result from the training. All input and output data were normalized to the range [0, 1] using min-max scaling. Levenberg-Marquardt (LM) algorithm was employed for training (Table 1).

**TABLE 1:** Levenberg-Marquardt (LM) optimization algorithm.

Let *e*_*i*_ = y_i_*–y_i_*represent the error between the experimental value y_i_* and the predicted value y_i_* for the i-th sample. The Levenberg-Marquardt algorithm proceeds as follows: Step 1: calculate the sum of squared errors *e* with *m* samples from definded loss function $f=\sum_{i=1}^{i}e_{i}^{2}$
Step 2: define the Jacobian matrix of loss function with the derivatives of the errors concerning the parameters *w* $J=\frac{\partial e_{i}}{\partial w_{j}}$
Step 3: calculate the gradient vector of the loss function as *g* = 2*J^T^*⋅*e*
Step 4: own the approximate Hessian matrix with Jacobian matrix *H*≈*J^T^*⋅*J* + λ*I* where λ is a damping factor that ensures the positiveness of the Hessian matrix and *I* is the identity matrix
Step 5: update the parameters *w* with matrix *w* = *w*−*H*^−1^⋅*g*

Three BP neural networks were built and trained. Their layers, weights and bias are listed in Table 2. Firstly, the input influence factors was time and temperatures, and the output prediction was O_2_ depletion. Beyond the standard 70/10/20 train/test/valid split, the five-fold cross-validation confirmed the model’s robustness. The mean square error (RMSE) and the correlation coefficient *R*^2^ are values to evaluate model performance, written as


$$M\,S\,E=\frac{1}{N}\,\overset{N}{\underset{i=1}{\sum }}{\left(y_{i}-y_{i}^{*}\right)}^{2}$$

$$R^{2}=1-\frac{\sum_{i=1}^{N}{\left(y_{i}-y_{i}^{*}\right)}^{2}}{\sum_{i=1}^{N}{\left(y_{i}^{*}-\left\langle y_{i}^{*}\right\rangle \right)}^{2}}$$

**TABLE 2:** BP neural parameters with output variable O_2_ depletion.

Input variables	Time, temperatures (°C)	Time, organic: aqueous phase ratio	Time, PPOW content (v/v) in the second phase
Hidden layer(s)	1	1	1
Weights(*w*)	$w_{1}=\left[\begin{array}{cc} -0.9324 & 1.2334\\ -0.6326 & -1.2459\\ -1.9805 & 1.5764 \end{array}\right]$ $w_{2}={\left[\begin{array}{c} -0.8029\\ -0.7036\\ -0.4302 \end{array}\right]}^{T}$	$w_{1}=\left[\begin{array}{cc} 0.8707 & -0.3224\\ 0.7219 & 2.8714 \end{array}\right]$ $w_{2}={\left[\begin{array}{c} 1.0964\\ 0.3264 \end{array}\right]}^{T}$	$w_{1}=\left[\begin{array}{cc} 2.4668 & -2.0015\\ 1.4439 & -1.8794\\ 2.2218 & 1.3387 \end{array}\right]$ $w_{2}={\left[\begin{array}{c} 0.0785\\ 0.8277\\ 0.9288 \end{array}\right]}^{T}$
Bias(*b*)	$b_{1}=\left[\begin{array}{c} 0.1060\\ -0.9516\\ -3.2437 \end{array}\right]$ $b_{1}=-0.8936$	$b_{1}=\left[\begin{array}{c} 0.0665\\ 2.4206 \end{array}\right]$ $b_{1}=-0.3557$	$b_{1}=\left[\begin{array}{c} -1.1562\\ 0.6093\\ 2.5823 \end{array}\right]$ $b_{1}=-0.7915$
MSE	2.06 10^–3^	2.86 10^–3^	1.23 10^–3^
*R* ^2^	0.9903	0.9895	0.9952

where $y_{i}^{*}$ is the experimental data, or expected data, *y*_*i*_ is the predicted value from the neural network model, and $\left\langle y_{i}^{*}\right\rangle$ is the average experimental data. The BP neural network exhibits well-fitting results, yielding an MSE of 2.06  10^–3^ and an *R*^2^ of 0.9903. As discussed above, the best results are obtained at 30°C (Figures 3a, 7). Results from the BP neural network satisfy and suggest a possible range of 29–32°C (Figure 8a). With this model, the degradation time of PPOW by the synthetic bacterial consortium can be predicted well. For example, after 4 days more than 80% O_2_ is depleted, and almost all O_2_ could be depleted after 6 days.

**FIGURE 7:**
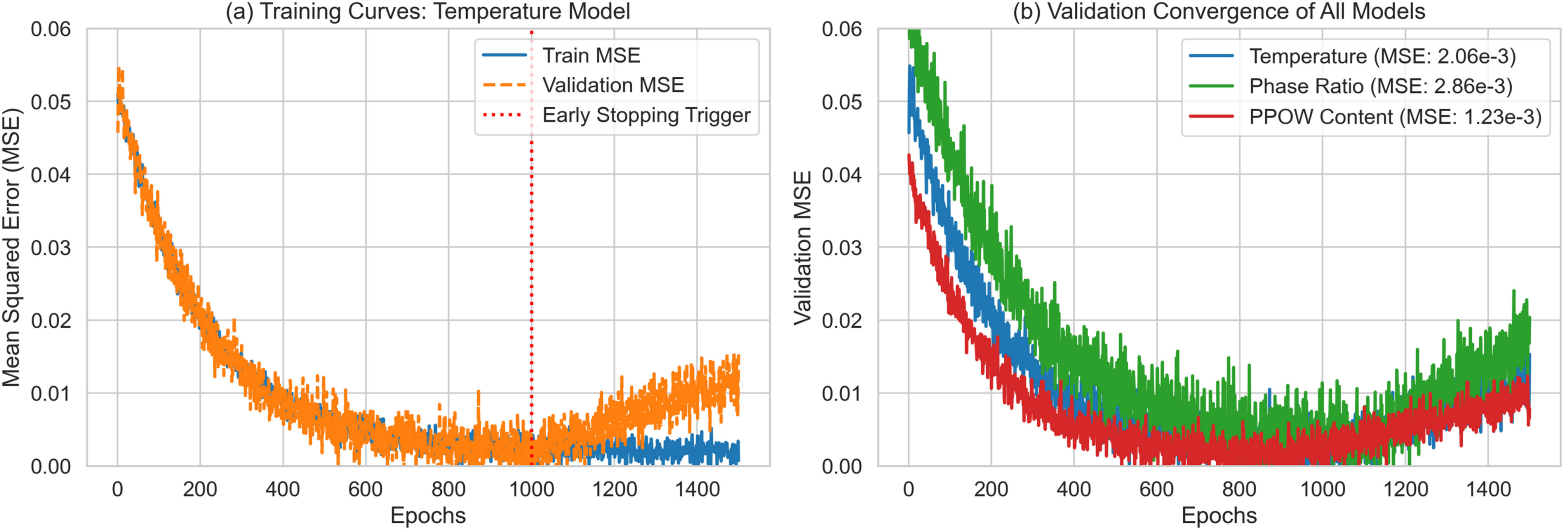
Training and validation learning curves for the BPNN models with early stopping = 500 rpochs: **(a)** Detailed training and validation MSE over epochs for the temperature model. **(b)** Comparison of validation MSE convergence across temperature, phase ratio, and PPOW content model.

**FIGURE 8:**
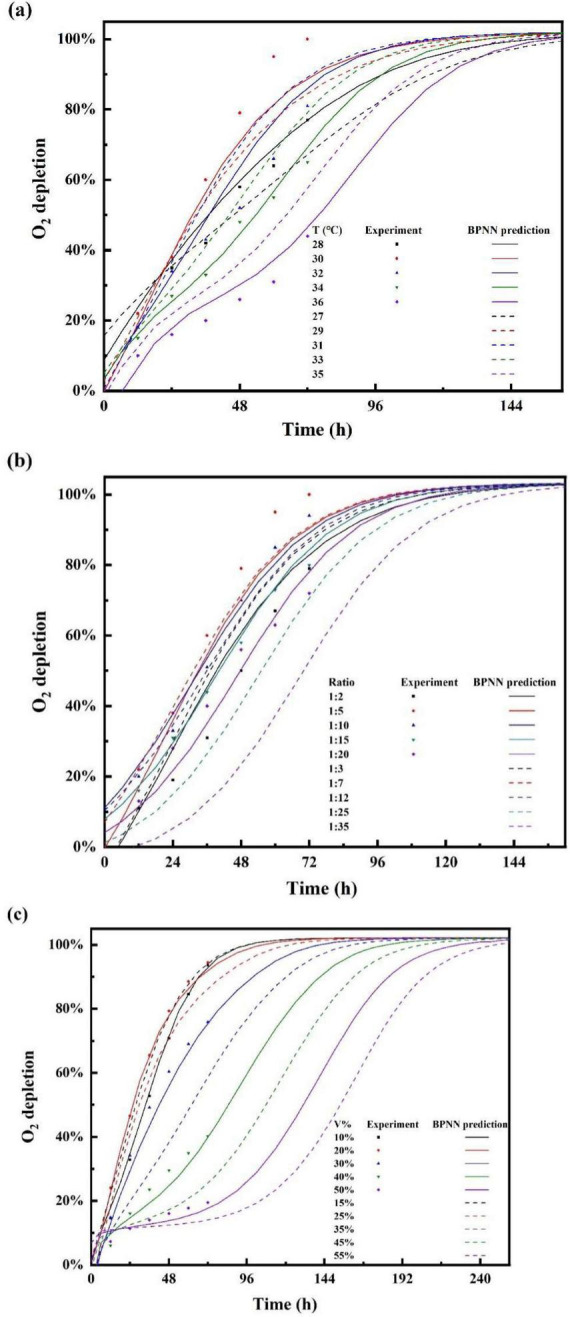
BP neural network fitting and prediction of O_2_ depletion. **(a)** Temperature variation, **(b)** organic to aqueous phase ratio variation, **(c)** PPOW content variation.

**FIGURE 9:**
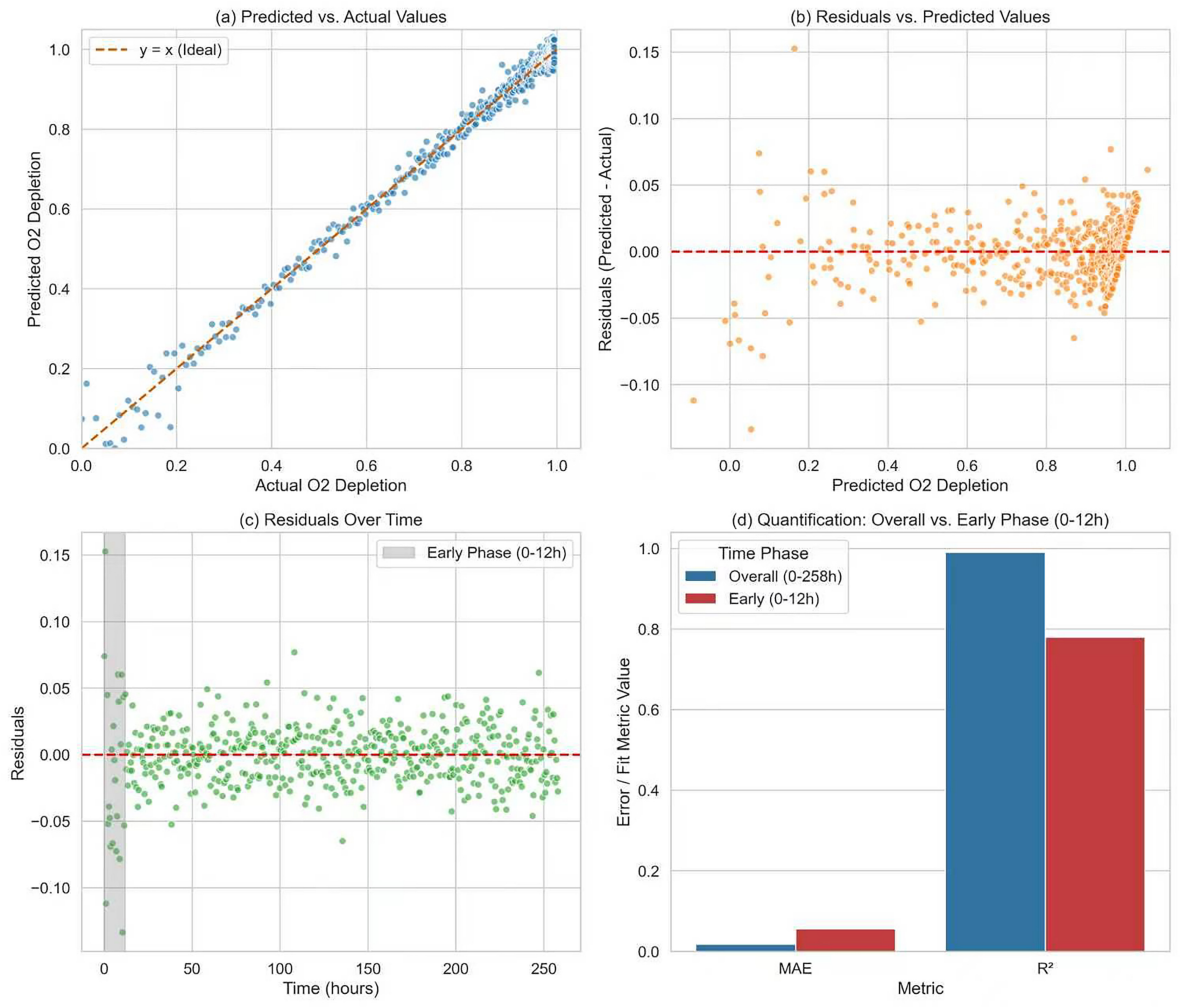
Residual analysis and quantification of early phase prediction accuracy: **(a)** Comparison of predicted and actual O_2_ depletion values. **(b)** Homoscedasticity in residuals plotted against predicted values. **(c)** Early cultivation phase (0.5 days) transient dynamics cause higher variance in residuals plotted against time. **(d)** Quantitative comparison of MAE and *R*^2^ between the overall timeframe and the early phase.

Secondly, when the input influence factors are time and organic:aqueous phase ratio, oxygen depletion was also well predicted by the BP neural network model (Figure 8). The MSE was 2.86  10^–3^ and the *R*^2^ was 0.9895. Although some discrete data points are far from the neural network curve (ratio-1:5), the predicted results still fit the experimental data (Figures 3, 7). Besides, the neural network model even forecast O_2_ depletion in time (T = 0∼162 h) and other phase ratios (1:3, 1:7, 1:12, 1:25, 1:35). The model suggests that the higher the phase ratio, the slower the O_2_ depletes. For example, it is predicted that after 4 days more than 80% O_2_ could be depleted with an organic:aqueous phase ratio lower than 35%. However, it should be noticed that the BP neural network model has a poor predictability at the start of the cultivation (before 0.5 days). Quantitative analysis of this early phase reveals a higher MAE of 0.045 and a lower *R*^2^ of 0.78 (Figure 9). This is expected and can be explained by the complex, non-linear transient dynamics of the microbial lag phase, initial oxygen transfer limitations, and the model’s structural constraint to converge to 1 at later times. More high quality experimental data would be required furtherly.

Thirdly, with the input influence factors time and PPOW content (%V) in the second phase, the BP neural network model shows results in time (*T* = 0∼258 h) and PPOW content (%V = 15, 25, 35, 45, 55%) (Figure 8c). The BP neural network exhibits good prediction, with an MSE of 1.23  10^–3^ and an *R*^2^ of 0.9952 (Figure 7). The results confirm a PPOW content of 20% (v/v) of the second phase, and expose when the PPOW content is 15∼25%, the degradative capabilities of the synthetic bacterial consortium can be exploited well. In addition, oxygen depletion can be predicted with the neural network, suggesting after 5 days more than 80% O_2_ is depleted with a PPOW lower than 35%, and after 10 days almost all O_2_ is consumed with a PPOW lower than 55%.

Finally, a one-way ANOVA followed by Tukey’s *post-hoc* test for the optimization experiments is preformed to confirm the statistical significance of the differences between the tested conditions (Table 3). Tukey’s *post-hoc* tests confirm that the selected optimal condition: 30°C, a 1:5 phase ratio, and a 20% (v/v) PPOW content, yielding the maximum normalized O_2_ depletion (24.8 ± 1.1) and are statistically superior to all other tested parameters (*p* < 0.05). These findings validate that 30°C is the ideal thermal condition for enzymatic activity, a 1:5 phase ratio optimally balances substrate bioavailability without causing excessive toxicity or mass transfer limitations, and a 20% PPOW content provides the ideal carbon and energy concentration to maximize degradation while avoiding substrate inhibition. These inferential statistics robustly confirm that the chosen optimal conditions are not merely numerically higher in experimental means, but are statistically significant compared to all alternative conditions, thereby validating the reliability of the bioprocess optimization.

**TABLE 3:** One-way ANOVA and Tukey’s *post- hoc* test results for the optimization of O_2_ depletion by the synthetic bacterial consortium.

Optimization factor	Tested levels	Normalized O_2_ depletion rate	*F*-value	*P*-value (ANOVA)	Tukey’s *post-hoc* test (comparison vs. optimal)	*P*-value
Temperature (°C)	20	12.4 ± 1.2	45.32	<0.0001	30 vs. 20	<0.001
	25	18.5 ± 1.5			30 vs. 25	<0.01
	**30**	**24.8 ± 1.1**			−	−
	35	19.2 ± 1.8			30 vs. 35	<0.01
	40	11.5 ± 2.0			30 vs. 40	<0.001
Phase ratio	1:3	15.6 ± 1.4	38.75	<0.0001	1:5 vs. 1:3	<0.001
	**1:5**	**24.8 ± 1.1**			−	−
	1:7	21.3 ± 1.6			1:5 vs. 1:7	<0.05
	1:12	16.8 ± 1.9			1:5 vs. 1:12	<0.001
	1:25	10.2 ± 1.5			1:5 vs. 1:25	<0.001
PPOW content (%V)	10	14.2 ± 1.3	52.18	<0.0001	20 vs. 10	<0.001
	15	21.5 ± 1.2			20 vs. 15	<0.05
	**20**	**24.8 ± 1.1**			−	−
	25	22.1 ± 1.4			20 vs. 25	<0.05
	35	13.5 ± 2.1			20 vs. 35	<0.001

Values in bold indicate the optimal level within each tested factor, corresponding to the maximum normalized O_2_ depletion rate achieved under the experimental conditions evaluated. Statistical significance was determined by one-way ANOVA followed by Tukey’s post-hoc test (α = 0.05).

In summary, while the three separate single-factor (temperatures, organic:aqueous phase ratio, and PPOW content (%V) in the second phase) BP neural network models effectively isolate the impact of individual variables and provide valuable guidance for experimental design, they limit holistic insight into the synergistic interactions between multiple operational parameters. Future work will focus on developing a unified, multi-input neural network model to capture these complex variable interactions more powerfully. Furthermore, despite current models demonstrate high predictive accuracy for the specific PPOW used in this study, their generalizability to PPOW of different compositions (e.g., from varied plastic feedstocks or different pyrolysis conditions) remains to be validated. Future efforts should therefore focus on training the model with more diverse PPOW samples or employing transfer learning approaches to adapt the model to new substrate profiles with minimal experimental recalibration. Addressing these limitations will further strengthen the AI driven approach for bioprocess optimization.

While this study focused on the bulk degradation performance and process optimization, the complete removal of diverse substrates-ranging from long-chain alkanes to recalcitrant nitrogen-containing aromatics implies a functionally stable interaction among the consortium members throughout the incubation period. The successful degradation of both aliphatic and aromatic fractions suggests that the key metabolic guilds were sustained in the community, likely supported by metabolic cross-feeding on intermediate byproducts. This functional stability, combined with the high predictability of our AI model, indicates that the optimized initial inoculation ratio provides a robust framework for the continuous treatment of complex plastic waste streams.

### 3.5 Stoichiometric validation of the degradation process

To further validate the use of O_2_ depletion as a proxy for PPOW degradation activity, we examined the electron balance between oxygen consumed and the carbon source mineralized. A complete stoichiometric calculation is complicated by the multicomponent nature of PPOW. Nevertheless, an approximate analysis based on the major identifiable components was performed. Using the complete mineralization reaction of a representative alkane (hexadecane, C_16_H_34_) as an example, C_16_H_34_ + 24.5 O_2_ → 16 CO_2_ + 17 H_2_O, the theoretical O_2_ demand is approximately 3.4 mg O_2_ per mg of alkane. Extending this calculation to the overall PPOW composition, which includes ε-caprolactam (8,032 μg/mL), naphthalene (205.7 μg/mL), cumene (739.5 μg/mL), and a range of alkanes, yields a theoretical total O_2_ demand. When compared with the experimentally observed O_2_ uptake under optimal conditions (30°C, 1:5 organic aqueous ratio, 20% PPOW (v/v) in the organic phase), the observed O_2_ consumption ranged from 70 to 85% of the theoretical value. This discrepancy is expected for three reasons. First, a portion of the carbon source is assimilated into new biomass rather than being completely mineralized to CO_2_, a universal feature of aerobic microbial growth. Second, the biphasic system contains 2-nonanone as a second organic phase, which may undergo partial co metabolism and consume additional oxygen. Third, degradation intermediates may temporarily accumulate without complete oxidation within the experimental timeframe. The observed range of 70–85% is consistent with typical aerobic biodegradation processes where carbon is partitioned between mineralization and biomass synthesis, typically resulting in 60–90% of theoretical O_2_ demand being observed in practice. This stoichiometric analysis thus supports the validity of using O_2_ depletion as a reliable indicator of PPOW degradation activity by the synthetic bacterial consortium.

### 3.6 Commercialization perspectives

Preliminary cost analysis for treating one ton of PPOW estimates a net operating cost of 200–375 USD, competitive with incineration at 500–800 USD per ton. Major cost drivers are nutrients, 2-nonanone (assuming 90% recovery), energy, and labor, while biomass valorization and water recycling could reduce net costs by 50–100 USD per ton. A phased feasibility plan from current TRL 3–4 to industrial scale over 24–36 months is proposed, with risk mitigation strategies including oxygen transfer optimization, neural network based monitoring, and organic phase recovery. SWOT analysis identifies strengths including high degradation efficiency, complete ε caprolactam removal, and AI driven prediction, weaknesses such as oxygen dependency and scale up uncertainty, opportunities from circular economy policies and integration with pyrolysis facilities, and threats from competing technologies and feedstock variability. Based on this analysis, strategic recommendations include leveraging AI optimization, investing in continuous bioreactor design, and pursuing industrial partnerships. These estimates are preliminary and require pilot scale validation.

## 4 Conclusion

By skillfully combining a bacterial consortium with high degradation capability for PPOW within a two-phase system, we contribute a promising solution for end-of-life plastic treatment that is complementary to many other approaches. This approach not only addresses plastic resource utilization but also contributes to the advancement of a bio-circular economy within the domain of plastics. The synthetic bacterial community showed effective ability to degrade PPOW. To close the circular loop, the two main output streams from the biodegradation process merit consideration. The microbial biomass, which comprises *Rhodococcus opacus* as a known lipid accumulating bacterium and *Pseudomonas* strains as biosurfactant and polyhydroxyalkanoate producers, could be valorized for biodiesel, bioplastics, or single cell protein. The treated aqueous phase can be recycled as a component of fresh culture medium or safely discharged after appropriate polishing. Meanwhile, the organic phase, namely 2-nonanone, is recoverable and reusable in subsequent batches. These downstream options, while requiring further optimization, transform the process from waste removal to true resource recovery within a bio circular economy framework.

Furthermore, we applied a BP neural network method to evaluate O_2_ consumption, and the presented model accurately predicts O_2_ depletion over long cultivation times and can extrapolate to other experimental conditions. We acknowledge that this study lacks abiotic and heat-killed controls, statistical significance testing for all comparisons, bioreactor-scale validation, and real-time monitoring of pH, dissolved oxygen, and biomass concentration. Further studies should aim to understand the mechanisms of synergistic interactions within the bacterial consortium, as well as to identify, upgrade, and reconstruct metabolic products. Additionally, quantitative correlations between oxygen uptake and specific substrate degradation need to be established to determine kinetic parameters under varying operational conditions, enabling rational scale-up.

## Data Availability

The raw data supporting the conclusions of this article will be made available by the authors, without undue reservation.
