# Supplementary material for: Synthetic bacterial consortium for degradation of plastic pyrolysis oil waste: experimental optimization and neural network modeling
Source: Front Microbiol. 2026 Jul 3;17:1817874. doi: 10.3389/fmicb.2026.1817874 (PMC13375889; doi:10.3389/fmicb.2026.1817874)
Supplement: Supplementary file 1 [file Data_Sheet_1.docx]

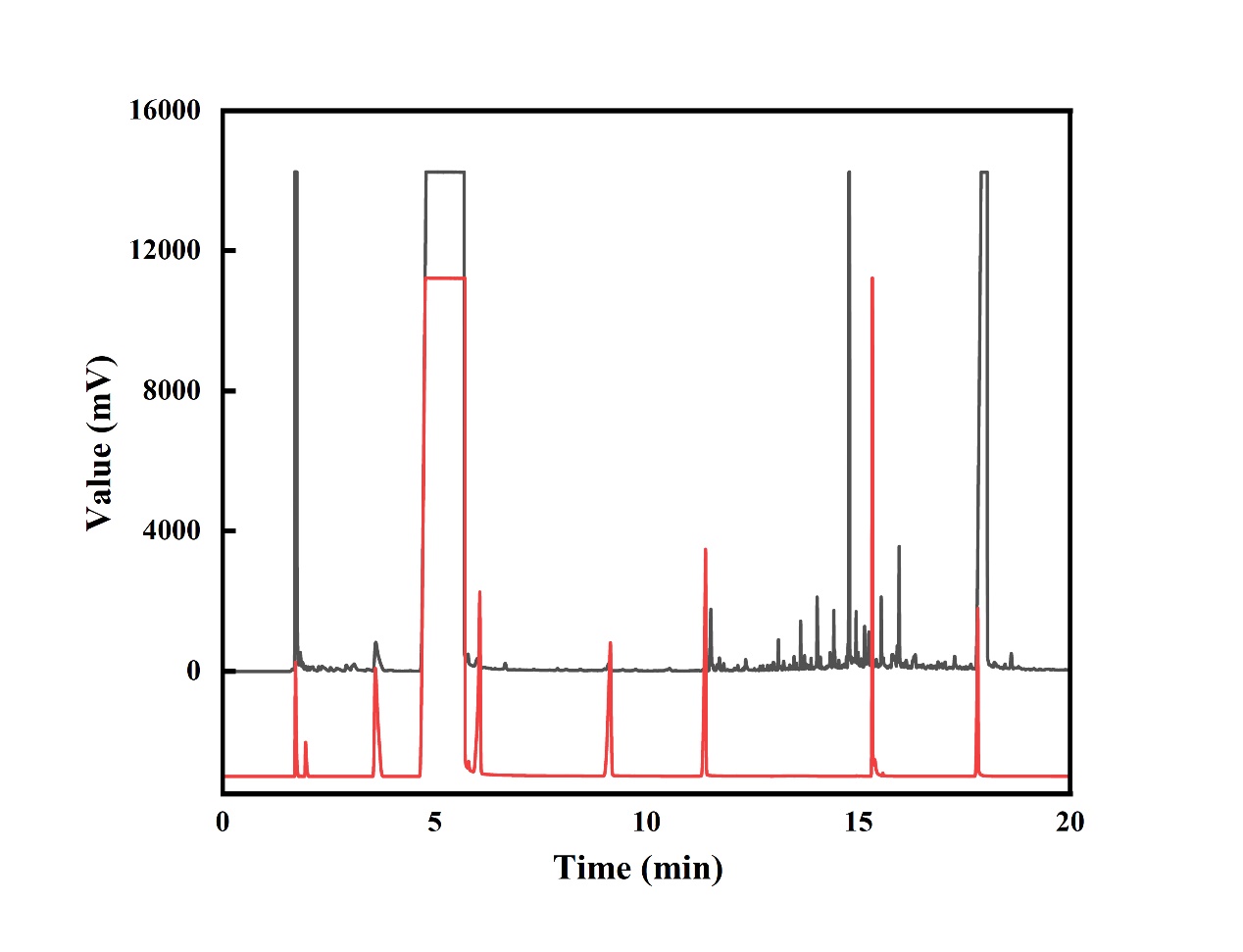
**
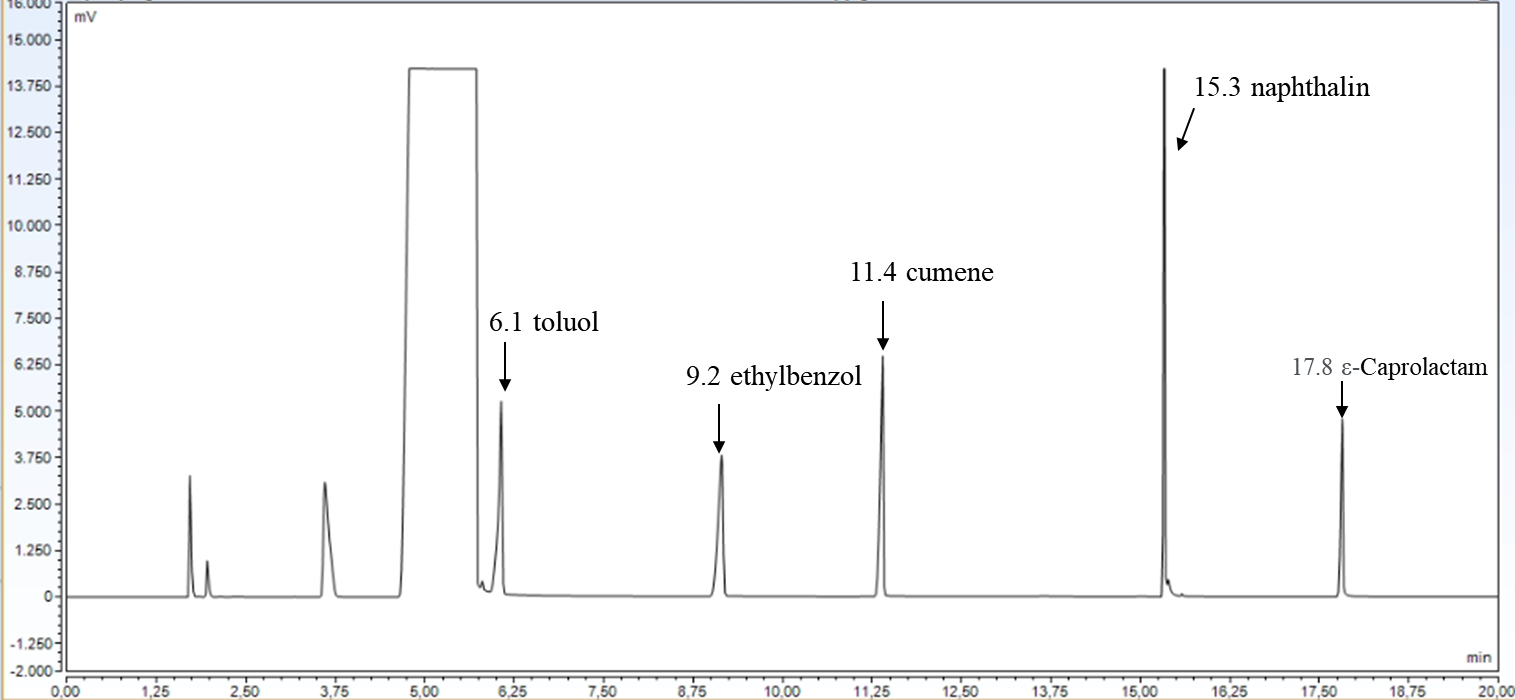
Fig S1. GC-FID chromatogram of PPOW(black) after filtered and diluted 10 times by n-hexane and standard samples of benzol, toluol, ethylbenzol, cumene, naphthalin and ε-caprolactam(red, from left to right).**

**Fig S2. Cumene,toluol,ethylbenzol, benzol, naphthalin and ε-caprolactam calibration curves by GC-FID.**


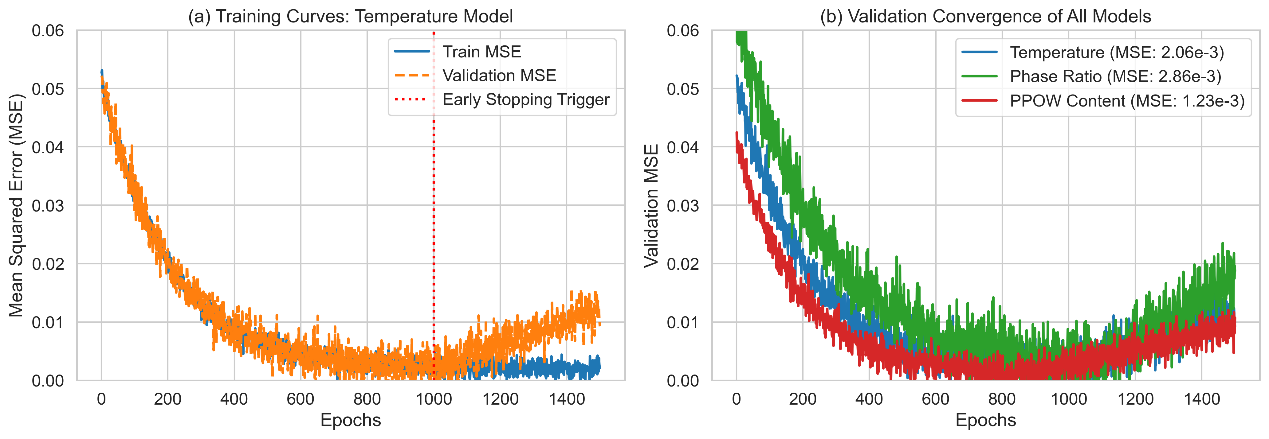


**Supplementary Fig. S3.** Training and validation learning curves for the BPNN models to confirm stability of our training process (early stopping = 500 rpochs): **(a)** Detailed training and validation MSE over epochs for the Temperature model. **(b)** Comparison of validation MSE convergence across Temperature, Phase Ratio, and PPOW Content model.


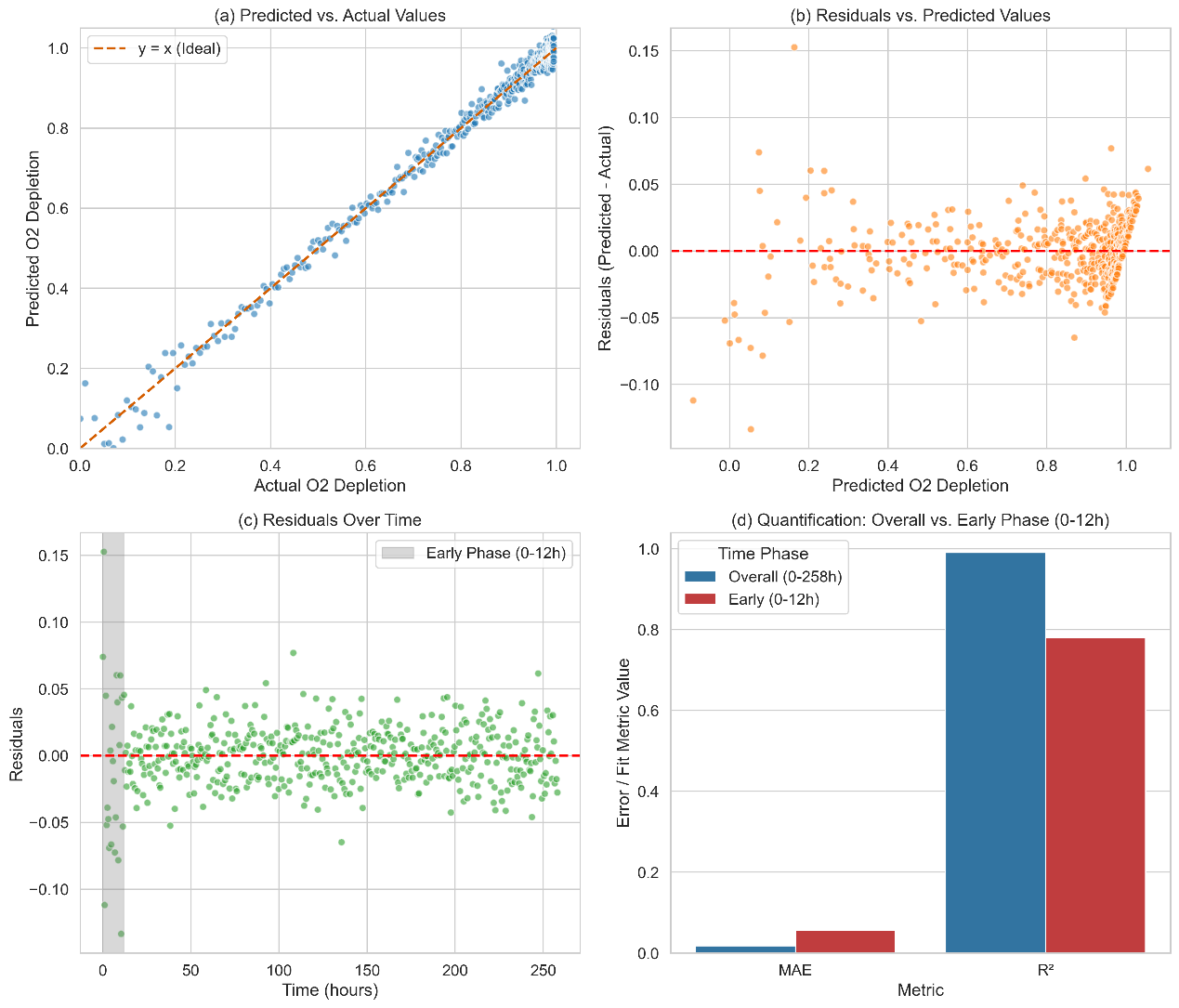


**Supplementary Fig. S4.** Residual analysis and quantification of early-phase prediction accuracy: **(a)** Comparison of predicted and actual O₂ depletion values. **(b)** Homoscedasticity in residuals plotted against predicted values. **(c)** Early cultivation phase (0.5 days) transient dynamics cause higher variance in residuals plotted against time. **(d)** Quantitative comparison of MAE and R² between the overall timeframe and the early phase.


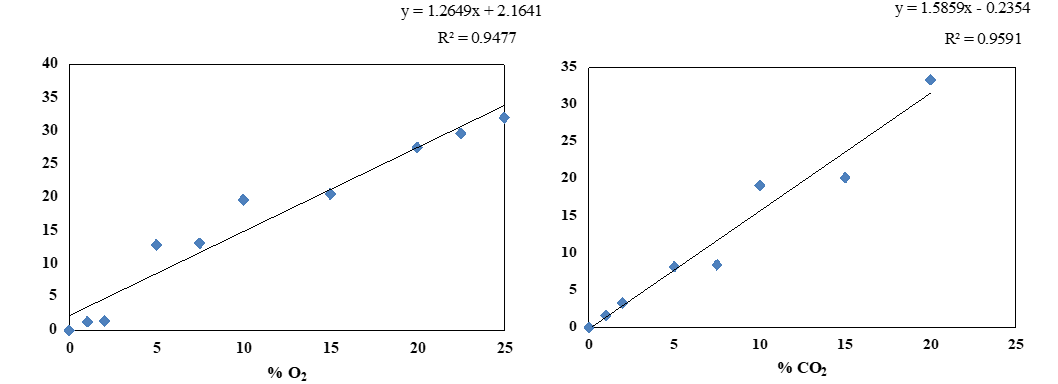


**Fig S5. CO_2_ and O_2_ calibration curves by gas chromatograph.**


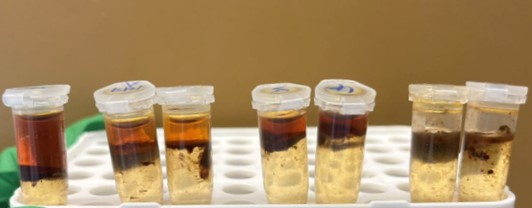


**Fig S6. PPOW after treated by different strains compounding ratios of *Rhodococcus opacus* DSM 43250: *Pseudomonas putida* KT2440: *Pseudomonas* sp*.* VLB120. From left to right:NC (negative control groups without adding** **bacteria, 7 days), 1:1:2 (3 days), 1:1:2 (7 days), 2:1:1 (3 days), 2:1:1 (7 days), 1:1:1 (7 days), 1:1:2 (7 days) at 30℃ with a second phase-water ratio of 1:5 and 20%(** **%V) of PPOW content in second phase.**

**Table S1. Concentrations of several major pollutants other than alkenes in PPOW**

| Name | Cumene | Toluol | Ethylbenzol | Naphthalin | ε-Caprolactam |
| --- | --- | --- | --- | --- | --- |
| Content (μg/mL) | 739 | 545 | 286 | 205 | 8032 |

**Table S2. One-way ANOVA and Tukey’s post-hoc test results for the optimization of O₂ depletion by the synthetic bacterial consortium.**

| **Optimization Factor** | **Tested Levels** | **Normalized O₂ Depletion Rate** | **F-value** | **p-value (ANOVA)** | **Tukey’s Post-hoc Test (Comparison vs. Optimal)** | **p-value** |
| --- | --- | --- | --- | --- | --- | --- |
| Temperature (°C) | 20 | 12.4 ± 1.2 | 45.32 | < 0.0001 | 30 vs. 20 | < 0.001 |
|  | 25 | 18.5 ± 1.5 |  |  | 30 vs. 25 | < 0.01 |
|  | **30** | **24.8 ± 1.1** |  |  | - | - |
|  | 35 | 19.2 ± 1.8 |  |  | 30 vs. 35 | < 0.01 |
|  | 40 | 11.5 ± 2.0 |  |  | 30 vs. 40 | < 0.001 |
| Phase Ratio | 1:3 | 15.6 ± 1.4 | 38.75 | < 0.0001 | 1:5 vs. 1:3 | < 0.001 |
|  | **1:5** | **24.8 ± 1.1** |  |  | - | - |
|  | 1:7 | 21.3 ± 1.6 |  |  | 1:5 vs. 1:7 | < 0.05 |
|  | 1:12 | 16.8 ± 1.9 |  |  | 1:5 vs. 1:12 | < 0.001 |
|  | 1:25 | 10.2 ± 1.5 |  |  | 1:5 vs. 1:25 | < 0.001 |
| PPOW Content (%V) | 10 | 14.2 ± 1.3 | 52.18 | < 0.0001 | 20 vs. 10 | < 0.001 |
|  | 15 | 21.5 ± 1.2 |  |  | 20 vs. 15 | < 0.05 |
|  | **20** | **24.8 ± 1.1** |  |  | - | - |
|  | 25 | 22.1 ± 1.4 |  |  | 20 vs. 25 | < 0.05 |
|  | 35 | 13.5 ± 2.1 |  |  | 20 vs. 35 | < 0.001 |
